# First renal metastasis report from tongue cancer

**DOI:** 10.1016/j.bjorl.2021.03.007

**Published:** 2021-04-08

**Authors:** Mustafa Korkmaz, Melek Karakurt Eryılmaz, Mustafa Karaağaç, Mehmet Artaç

**Affiliations:** Necmettin Erbakan University, School of Medicine, Department of Medical Oncology, Konya, Turkey

## Introduction

Tongue cancer (TC) is in the oral cavity cancers subgroup of head and neck cancers (HNC). Squamous cell carcinoma (SCC) is the predominant histological type that occurs in the TC group. The incidence of tongue SCC (TSCC) is increasing and has a relatively poor prognosis and aggressive clinical behavior.^1^ The average age of TSCC at the time of diagnosis is 61 years. Despite an increasing trend in the prevalence of TSCC, only about 2% of patients are diagnosed before age 35 and 7% before age 45.^2^

The kidney is a rare site of metastasis. Renal metastases are frequently reported in autopsy series (7%–12%), but are rare in clinical practice.^3^ Renal metastases mainly originate from the lung, breast, digestive organs (especially esophagus, stomach and colon), and melanoma.^4^

In this report, we present a young tongue cancer patient who developed renal metastasis and review the literature on non-nasopharyngeal head and neck cancers with renal metastasis.

## Case report

**Figure 1:**
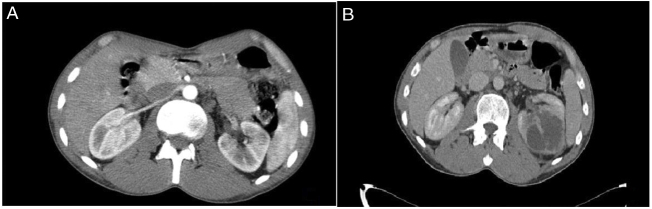
(A) Abdominal CT scan in at the time of diagnosis, (B) Abdominal CT scan axial view of left renal mass. A solid expansive lesion of the medial pole of the left kidney, with the presence of bilobed hypodense area of cystic appearance approximately 40 × 24 mm in the renal parenchyma.

In April 2013, a 36-year-old male patient presented with a wound on the tongue and swelling in the neck. He had been smoking 1 pack of cigarettes a day for 15 years and did not drink alcohol. Physical examination revealed an ulcerated 30 × 20 mm (mm) tumor on the right side of the tongue and palpable lymph nodes on the right side of the neck. An incisional biopsy was performed from the tongue lesion and its pathology was reported as TSCC. In contrast-enhanced neck magnetic resonance imaging (MRI), malignant lymphadenopathies in the right cervical 2A region were reported, and no metastases were observed on computed tomography (CT) of the chest, abdomen, and pelvis. Right hemiglosectomy plus bilateral neck dissection was performed in August 2013. The pathology result was grade 1 TSCC. The tumor size was 30 × 25 mm, the depth of the tumor was 10 mm, and three of the 48 lymph nodes had metastases. There was no capsule invasion, extranodal extension (ENE), perineural or lymphovascular invasion. Surgical margins were reported as negative. Positive lymph nodes were right- sided and the size of the largest metastatic lymph node was 20 mm. He was staged as pT2 pN2b M0, stage IVA (American Joint Committee on Cancer (AJCC) seventh edition). He received adjuvant therapy with concurrent cisplatin and radiotherapy. In his followup in April 2014, a 40 × 24 mm hypodense cystic lesion was observed in the middle of the left kidney (Fig. 1). In positron emission computed tomography-CT (PET-CT), no additional involvement was observed. The case was evaluated as compatible with metastasis at the tumor board, and renal mass biopsy was performed. Pathology of renal mass biopsy was reported as SCC, head and neck tumor metastasis. Left total nephrectomy was performed in the patient who had no other distant metastasis. Nephrectomy pathology was consistent with grade 1 SCC, tumor size was 80 mm, no perineural and lymphovascular invasion, and surgical margins were clear. After nephrectomy, 4 cycles cisplatin plus fluorouracil was given. In September 2015, multiple metastases were detected in the patient's lungs and bones. After 3 cycles of carboplatin plus docetaxel, the patient, who did not want to receive chemotherapy, was followed up with the best supportive care (BSC). He died on January 2016.

## Methods

We searched the PubMed and Google Scholar databases from their earliest records until December 20, 2020, reviewed case reports and literature reviews of renal metastases from head and neck cancer.

## Discussion

Distant metastasis rates in HNC have been reported to be approximately 15%–25% of patients. Advanced T stages in cancers are associated with the highest incidence of distant metastasis. The most common site of metastasis in head and neck SCC is pulmonary metastasis, with 66%, and other metastatic sites are bone, liver, skin, mediastinum and bone marrow.^5^ Renal metastasis is a rare event. It is mostly based on autopsy series with statistical data. According to autopsy reports, the incidence of renal metastasis is 3–15%, and this is mainly due to lung, breast, gastric and colon cancers and melanoma.^6^ In clinical practice, renal metastases of HNC are extremely rare, but a few cases of HNC that developed renal metastasis have been reported. Renal metastasis has been reported from 4 laryngeal cancers and 1 hypopharyngeal cancer.^7^, ^8^ The characteristics of these cases are summarized in Table 1. In a retrospective study evaluating 151 renal metastasis patients whose primary tumor was not renal cell carcinoma, HNC was the primary tumor site in 9 (6%) patients. The location of the head and neck region of these 9 HNC patients was not specified in this study. Nephrectomy was performed in 48 (31.8%) patients, 9 of which were partial (18.8%) and 39 radicals (81.2%) and ablation was performed in 3 (2%) patients. The remaining 100 (66.2%) patients were followed up with palliative treatment without surgery.^9^ In another retrospective analysis of 43 patients with renal metastases, the primary tumor site was HNC in 4 patients. Subtypes were Hurthle cell thyroid carcinoma in 2 patients, papillary thyroid carcinoma in 1 patient, and ex-pleomorphic adenoma of the salivary gland in 1 patient. The median renal metastasis development interval was 3.1 years, but this range was wide (0–21.6 years), and the first distant metastasis site detected in 37% of cases was renal. Most of the patients (56.7%) were completely asymptomatic. In addition, most of renal metastases were solitary and the median tumor diameter was 4.1 cm. The median overall survival (OS) for the whole study population from the time of diagnosis of renal metastases is 1.13 years. The median OS was better in patients who underwent kidney surgery compared to those without kidney surgery (2.24-years versus 0.72-years).^10^ There is no consensus on the treatment of kidney metastases. Each case should be evaluated within itself. Local treatments such as nephrectomy or stereotactic body radiation (SBRT) are options. Nephrectomy is recommended for patients who do not have distant metastases and whose primary disease is under control.^4^ In our case, renal metastasis developed 2 years after the diagnosis of TC and it was the first metastasis site. Left total nephrectomy was performed because there was no distant metastasis other than renal. The survival time of our patient after the diagnosis of renal metastasis was 1.58 years.

**Table 1:** Clinicopathological features of renal metastases from head and neck cancer cases.

Author, years	Patientof number	Age	Gender	Primary site	Histology	Timing of renal metastasis at diagnosis	Symptoms in the diagnosis of renal metastases	Treatment	Follow up
Wada et al. (2002)	1	67	Male	Hypopharyngeal carcinoma	SCC	1-years after	Gross hematuria and left flank discomfort	Radical left nephrectomy	He was still alive 8-months after left nephrectomy without gross hematuria or left flank discomfort
Del Vecchio et al. (2017)	1	63	Male	Laryngeal cancer	SCC	42-weeks	Right lower quadrant pain	Radical right nephrectomy	The patient was referred to a radiation oncologist to discuss the possibility of adjuvant radiotherapy
Paul et al. (1999)	1	42	Male	Laryngeal cancer	SCC	2-years after	Abdominal pain	Radical left nephrectomy	6-months following first evidence of distant metastasis
Lecoeuvre et al. (2003)	1	60	Male	Laryngeal cancer	SCC	4-years after	Renal colic	Carboplatin chemotherapy	The patient died 6-months after renal metastasis
Erbag et al. (2013)	1	55	Male	Laryngeal cancer	SCC	5-years after	N/A	Partial right nephrectomy	N/A
Our case	1	36	Male	Tongue Cancer	SCC	8-months after	No symptoms	Radical left nephrectomy	The patients died 20-months after renal metastasis

N/A, Not Available; SCC, Squamous Cell Carcinoma.

## Conclusion

We describe the renal metastasis report from TC. To the best of our knowledge, our case report is the first renal metastasis report of TC in a young patient described in the literature.

## Conflicts of interest

The authors declare no conflicts of interest.
